# Effects of Radiofrequency Catheter Ablation of Atrial Fibrillation on Soluble P-Selectin, Von Willebrand Factor and IL-6 in the Peripheral and Cardiac Circulation

**DOI:** 10.1371/journal.pone.0111760

**Published:** 2014-11-12

**Authors:** Jelena Kornej, Borislav Dinov, Andrew D. Blann, Sascha Rolf, Arash Arya, Josephine Schmidl, Daniela Husser, Gerhard Hindricks, Andreas Bollmann, Gregory Y. H. Lip

**Affiliations:** 1 University of Leipzig, Heart Center, Department of Electrophysiology, Leipzig, Germany; 2 University of Birmingham Centre for Cardiovascular Sciences, City Hospital, Birmingham, United Kingdom; University of Bologna, Italy

## Abstract

**Background:**

Catheter ablation (CA) of atrial fibrillation (AF) is associated with inflammatory response, endothelial damage and with increased risk of thrombosis. However, whether these processes differ in peripheral *and* cardiac circulation is unknown.

**Methods:**

Plasma markers (von Willebrand factor (vWf), soluble P-selectin (sPsel) and interleukin-6 (IL-6)) were measured by ELISA at three time points in 80 patients (62±10 years, 63% males, 41% paroxysmal AF) undergoing CA. These were at baseline – from femoral vein (FV) and left atrium (LA) before ablation; directly after ablation – from the pulmonary vein (PV), LA and FV; and 24 hours after procedure – from a cubital vein (CV).

**Results:**

The levels of vWF and IL6 – but not sP-sel – increased significantly 24h after procedure (p<0.001). Baseline vWF was significantly associated with persistent AF (Beta = .303, p = 0.006 and Beta = .300, p = 0.006 for peripheral and cardiac levels, respectively), while persistent AF (Beta = .250, p = 0.031) and LAA flow pattern (Beta = .386, p<0.001) remained associated with vWF in cardiac blood after ablation. Advanced age was significantly associated with IL6 levels at baseline and after ablation in peripheral and cardiac blood. There were no clinical, procedural or anti-coagulation characteristics associated with sP-sel levels in cardiac blood, while peripheral sP-sel levels were associated with hypertension before (Beta = −.307, p = 0.007) and with persistent AF after ablation (Beta = −.262, p = 0.020).

**Conclusions:**

vWF levels are higher in persistent AF and are associated with LAA rheological pattern after AF ablation. Increase of peripheral vWF and IL6 levels after procedure supports current AF ablation management with careful control of post-procedural anticoagulation to avoid ablation-related thromboembolism.

## Introduction

Atrial fibrillation (AF) is a major public health problem and its incidence increases as the population ages. AF is a pro-thrombotic and pro-inflammatory disorder leading to substantial mortality and morbidity due to severe cardio- and cerebrovascular complications. Catheter ablation has become a frequently performed procedure for symptomatic relief of this condition multiple procedures are required to achieve acceptable success rates [1].

An association between rhythm outcomes after catheter ablation and markers of inflammation is known [2], [3], [4]. Recently, we demonstrated that AF ablation leads to changes in inflammatory biomarkers that had an impact on AF recurrences during 6 months follow-up after procedure [3], [5]. Some studies analyzed acute changes in biomarker levels related to ablation itself. For example, soluble P-selectin (sP-sel, implying platelet activation) increased in cardiac circulation within minutes of AF onset [6], [7]. Furthermore, catheter ablation demonstrated effects on pro-coagulant state and endothelial dysfunction, with increased concentrations of tissue plasminogen activator and von Willebrand factor (vWF, implying endothelial damage) at 24 h after procedure [8]. However, immediate effects on biomarkers of endothelial function, pro-thrombotic and pro-inflammatory changes in patients with AF in peripheral *and* cardiac blood are still unexplored. Furthermore, recent studies have failed to compare directly responses in paroxysmal (PAF) versus persistent AF (PersAF).

While the changes in plasma marker profiles in *peripheral* blood are associated with ablation – that partly might be explained by a ‘wash-out’ phenomenon – and this association in cardiac blood is understudied. Therefore, we hypothesized that AF ablation leads to increase of pro-inflammatory, pro-thrombotic and markers of endothelial damage that is more presentable in cardiac than in peripheral blood. Furthermore, we hypothesized that these markers correlate significantly in both peripheral and cardiac circulation.

## Methods

### Study population

We recruited 80 patients with symptomatic AF who underwent AF catheter ablation at Heart Center Leipzig, Germany in October 2012 and January 2013. The study was approved by the local ethics committee (Medical Faculty, University Leipzig) and patients provided written informed consent for participation. Paroxysmal (PAF) and persistent AF (PersAF) were defined according to the current guidelines [9]. PAF was defined as self-terminating within 7 days after onset documented by previous ECG or Holter-ECG. PersAF was defined as an AF episode either lasting longer than 7 days or requiring drug or direct current cardioversion for termination. In all patients, transthoracic and transesophageal echocardiography were performed prior to the ablation. All class I or III antiarrhythmic medications with exception of amiodarone were discontinued for at least 5 half-lives before the AF ablation.

### Catheter ablation

Patients presenting with AF at the beginning of the procedure were electrically cardioverted and ablation was performed during sinus rhythm (i.e. AF termination with ablation was not attempted). Pulmonary vein (PV) isolation was performed by sequential application of radiofrequency energy at the antrum of the pulmonary veins. End-point was isolation of the PV with proof of both exit and entrance block. After the isolation of the pulmonary veins, electoanatomical voltage maps of the LA body in sinus rhythm were created in each patient using ablation catheter as a roving catheter. Potentials with amplitudes over 0.5 mV were defined as normal, and potentials under 0.2 mV as low-voltage. According to the underlying substrate and induced left atrial macro reentry tachycardias (LAMRT) additional lines transecting the scar areas to connect with healthy tissue or anatomical obstacles were ablated. Linear ablation of cavotricuspid isthmus was performed in cases of induction of typical atrial flutter after obtaining the blood samples from left atrium. Detailed ablation protocol is provided as **File S1**.

### Blood samples

Blood samples were obtained during catheter ablation and 24-(48) hours after procedure. All plasma markers were serially measured at three defined time points: baseline samples (blood from femoral vein (FV) and left atrium (LA) before first radiofrequency pulse) were taken immediately after obtaining the first access to the FV and immediately after trans-septal puncture (T1); at the end of the procedure further blood samples (left superior pulmonary vein (PV), LA and FV) were taken before the catheter and sheath removal (T2); the last sample was taken at the day after procedure from cubital vein (CV) (T3; 24 h). The blood from LSPV (in 41 patients, 51% study population) was taken to investigate the differences in plasma markers up- and downstream regarding the ablation place (LA). Because the patients were usually discharged 24 h after ablation, only in small part of study population (10 patients) the blood was taken 48 h after procedure (cubital vein, T4; 48 h).

All blood samples were centrifuged within 1 h of collection (3500×g for 10 min at 20°C) and plasma aliquots were stored at −70°C for subsequent analysis. Plasma levels of soluble P-selectin (sP-sel), interleukin-6 (IL-6, using a high-sensitivity kit) and von Willebrand factor (vWF) were quantified using commercially available specific enzyme-linked immunoabsorbent assays (ELISA) according to manufacturers protocols (R&D Systems (Abingdon, UK) for sP-sel and IL-6 (high sensitivity); Dako-Patts (Ely, UK) for vWF). The unit for vWF is IU/dL and was standardized with reference vWF from National Institute for Biological Standards and Controls (Potters Bar, Hertfordshire, UK). Intra-assay coefficients of variation for all ELISA assays were <5%, and inter-assay variances were <10%. Results were compared with standard curves and the lower detection limits were as follows: P-Selectin 125 pg/ml; high-sensitive IL-6 0.132 pg/ml; von Willebrand factor −5 IU/ml.

### Statistical analysis

Data are presented as mean and standard deviation (SD) for normally distributed or median [interquartile range] for skewed continuous variables and as proportions for categorical variables. Continuous variables were tested for distribution using the Kolmogorov-Smirnov test. The differences between continuous values were assessed using an unpaired t-test or the Mann–Whitney, and a chi-square test for categorical variables. Paired data was analysed by paired t-test or Wilcoxon's method. Comparison of markers from different sites was performed using repeated measures ANOVA test or by Friedman's method. Correlations were performed using Spearman's rank correlation method. Linear regression analysis was used to identify factors associated with plasma marker changes related to ablation. Multivariable analysis, which included variables with a p-value <0.2 found on univariable analysis, was performed to identify independent predictors of plasma marker changes.

A p-value <0.05 was considered as statistically significant, and all analyses were performed with SPSS statistical software version 17.

## Results

### Baseline plasma marker profiles

Baseline, echocardiographic and procedural characteristics of the study population are presented in **Table 1**. Transesophageal echocardiography was performed in sinus rhythm (40%) and AF (60%). Although patients with SR had better LAA blood flow than those with AF but these differences were not significant (0.61 [0.5–0.8] m/s versus 0.5 [0.4–0.7] m/s, p = 0.524).

**Table 1:** Clinical, echocardiographic and procedural characteristic of the study population.

	Study population	PAF	PersAF
	n = 80	n = 34	n = 46
Age, years	62±10	60±11	63±10
Males, %	62	62	65
Paroxysmal AF, %	41		
Re-do, %	35	38	35
RR systolic, mmHg	144±22	141±33	141±18
RR diastolic, mmHg	84±13	82±10	84±14
Heart rate, bpm	79±22	71±20	86±20
BMI, (kg/m^2^)	30±6	29±7	30±5
eGFR, ml/min/1.73 m^2^	97±31	96±28	99±34
**Medical history, %**			
Hypertension	85	77	89
Diabetes mellitus	19	9	26
Vascular disease	29	18	37
Previous TE	8	9	7
CHADS_2_ score	1 [1]–[2]	1 [1]–[2]	1 [1]–[2]
CHA_2_DS_2_-VASc score	2 [1]–[3]	2 [1]–[3]	3 [2]–[4]
**Medical treatment, %**			
Beta blockers	85	82	87
ACEI/ARB	63	50	74
Statins	34	21	41
**Anticoagulation, %**			
VKA	58	50	65
NOACs	28	24	33
**Echocardiographic data**			
LAD, mm	44±6	41±6	46±6
LV-EDd, mm	51±6	49±6	52±8
EF, %	56±10	59±8	54±10
**Procedural data**			
Substrate modification, %	32	18	46
CTI, %	27	21	28
Low voltage areals, %	34	27	41
CV during procedure, %	61	38	78
Heparin, total, IU	13,253±3,152	12,382±3,257	13,957±3,049
First ACT	268±60	259±56	271±67
ACT at the end of procedure	285±49	282±54	287±45
Ablation time, s	1,688 [1103–2,704]	1,278 [962–2,004]	2,109 [1,398–2,843]
Total ablation power, J	56,616 [39,009–90,262]	45,414 [35,061–61,952]	72,769 [44,587–93,872]
Procedure time, min	150 [120–213]	120 [106–175]	180 [148–233]

**Abbreviations**: BMI – body mass index; TE - thromboembolic events, VKA – vitamin K antagonists; ACEI – angiotensin converting enzyme inhibitors; ARB – angiotensin receptor blockers; non-OAC – anticoagulation with aspirin, clopidogrel or low molecular weight heparins, LAD – left atrial diameter, IVSd – interventricular septum end-diatolic, LV-EDd – left ventricular end-diastolic diameter, EF – ejection fraction, LAA – left atrial appendage, SR – sinus rhythm, CV – electrical cardioversion, ACT – activated clotting time.

Data mean ± SD, median [interquartile range], or actual numbers/%.

Using multivariable analysis, baseline vWF levels were significantly associated with persistent AF (Beta = .303, p = 0.006 and Beta = .300, p = 0.006 for peripheral and cardiac levels, respectively, **Table 2**), while advanced age was significantly associated with IL6 levels at baseline in peripheral and cardiac blood (**Table 3**). There were no clinical characteristics associated with sP-sel levels in cardiac blood, but peripheral sP-sel levels at baseline were associated with hypertension (Beta = −.307, p = 0.007) before ablation (**Table 4**). There were significant correlations between vWF and IL6 levels in peripheral (r^2^ = .251, p = 0.026) but not cardiac blood (r^2^ = .147, p = 0.196) before AF ablation.

**Table 2:** Association between clinical parameters and baseline (pre-ablation) von Willebrandt factor levels.

	vWF _FV_pre_				vWF _LA_pre_			
Variables	UV		MV		UV		MV	
	Beta	*p*-value	Beta	*p*-value	Beta	*p*-value	Beta	*p*-value
Age	−.099	0.386			.031	0.788		
Persistent AF	.317	0.004	.303	0.006	.323	0.004	.300	0.006
Female gender	.062	0.586			.069	0.547		
Hypertension	.043	0.708			.092	0.421		
Diabetes	.102	0.373			.029	0.796		
CAD/PAD	.004	0.971			.104	0.363		
BMI	.216	0.056	.163	0.162	.176	0.120	.156	0.147
eGFR	.148	0.192	.079	0.497	−.004	0.971		
LA diameter	.067	0.557			.074	0.518		
EF	−.141	0.216			.028	0.808		
Spontaneous contrast	.135	0.236			.176	0.121	.157	0.145
Oral anticoagulation	−.064	0.235			−.093	0.415		

**Table 3:** Association between clinical parameters and baseline (pre-ablation) interleukin-6 levels.

	IL6 _FV_pre_				IL6 _LA_pre_			
Variables	UV		MV		UV		MV	
	Beta	*p*-value	Beta	*p*-value	Beta	*p*-value	Beta	*p*-value
Age	.211	0.062	.241	0.029	.252	0.025	.279	0.011
Persistent AF	.134	0.239			.058	0.609		
Female gender	.101	0.376			.101	0.374		
Hypertension	.021	0.852			.083	0.469		
Diabetes	.065	0.568			.056	0.624		
CAD/PAD	−.055	0.632			−.057	0.618		
BMI	.201	0.075	.207	0.059	.201	0.076	.213	0.052
eGFR	−.042	0.714			−.059	0.606		
LA diameter	.133	0.243			.080	0.482		
EF	−.195	0.085	−.197	0.073	−.151	0.184	−.155	0.154
Spontaneous contrast	−.062	0.585			−.092	0.422		
Oral anticoagulation	−.018	0.872			.012	0.913		

**Table 4:** Association between clinical parameters and baseline (pre-ablation) soluble p-Selectin levels.

	sP-sel _FV_pre_				sP-sel _LA_pre_			
Variables	UV		MV		UV		MV	
	Beta	*p*-value	Beta	*p*-value	Beta	*p*-value	Beta	*p*-value
Age	−.153	0.179	−.077	0.487	−.120	0.293		
Persistent AF	−.124	0.278			−.236	0.036	−.209	0.063
Female gender	−.137	0.229			−.029	0.800		
Hypertension	−.315	0.005	−.307	0.007	−.206	0.068	−.174	0.121
Diabetes	.039	0.731			.034	0.768		
CAD/PAD	.035	0.758			.043	0.709		
BMI	−.090	0.429			.046	0.690		
eGFR	.128	0.261			.118	0.301		
LA diameter	.007	0.949			−.119	0.298		
EF	−.090	0.431			−.019	0.866		
Spontaneous contrast	−.166	0.143	−.186	0.086	.044	0.703		
Oral anticoagulation	.020	0.858			−.069	0.545		

### Effects of catheter ablation on plasma markers

**Figure 1:**
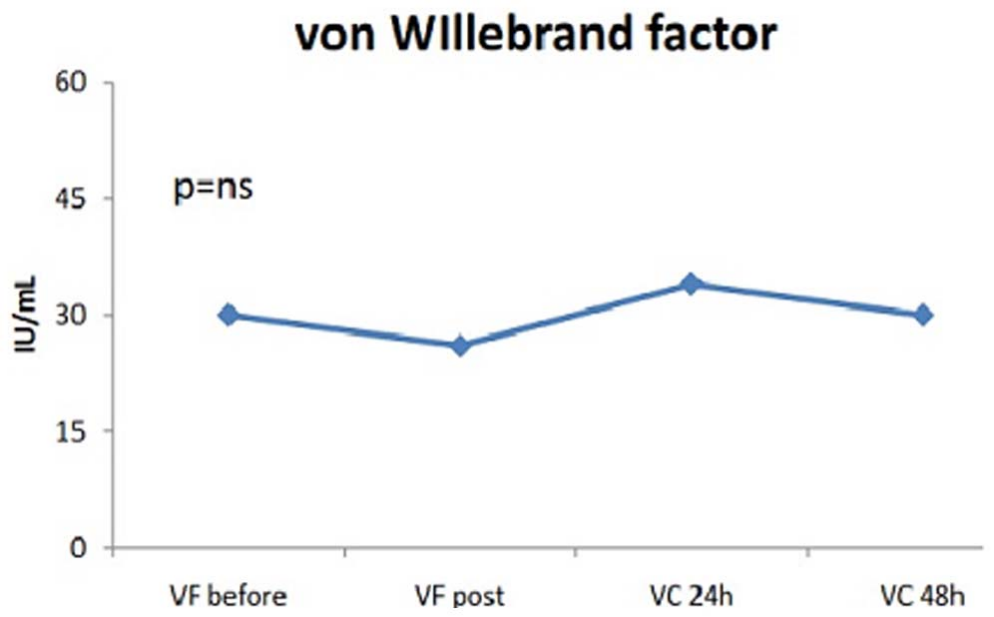
Changes in von Willebrand factor levels after catheter ablation.

**Figure 2:**
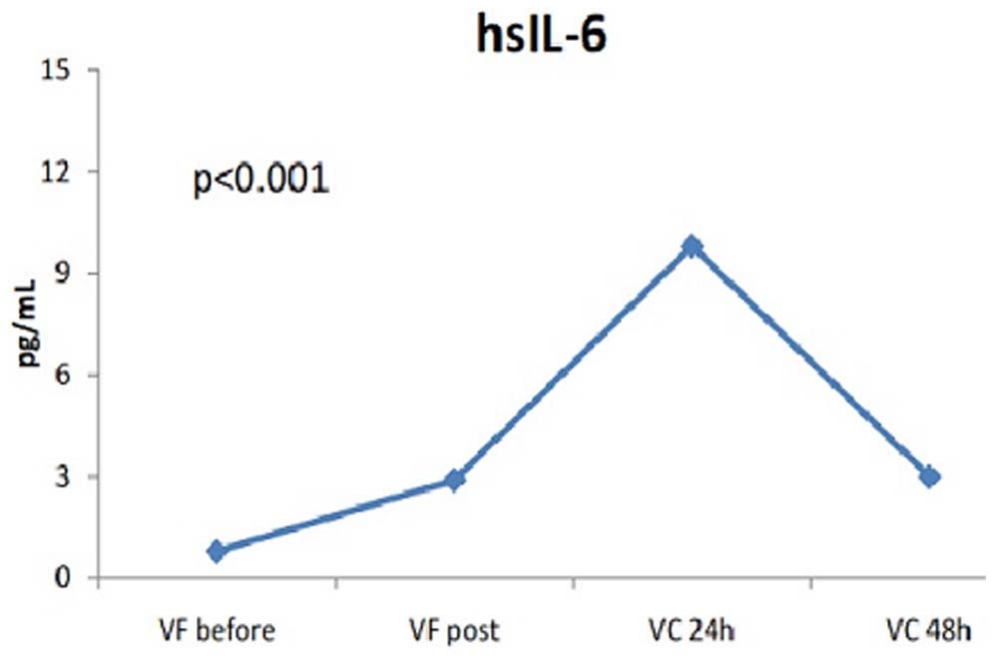
Changes in interleukin-6 levels after catheter ablation.

**Figure 3:**
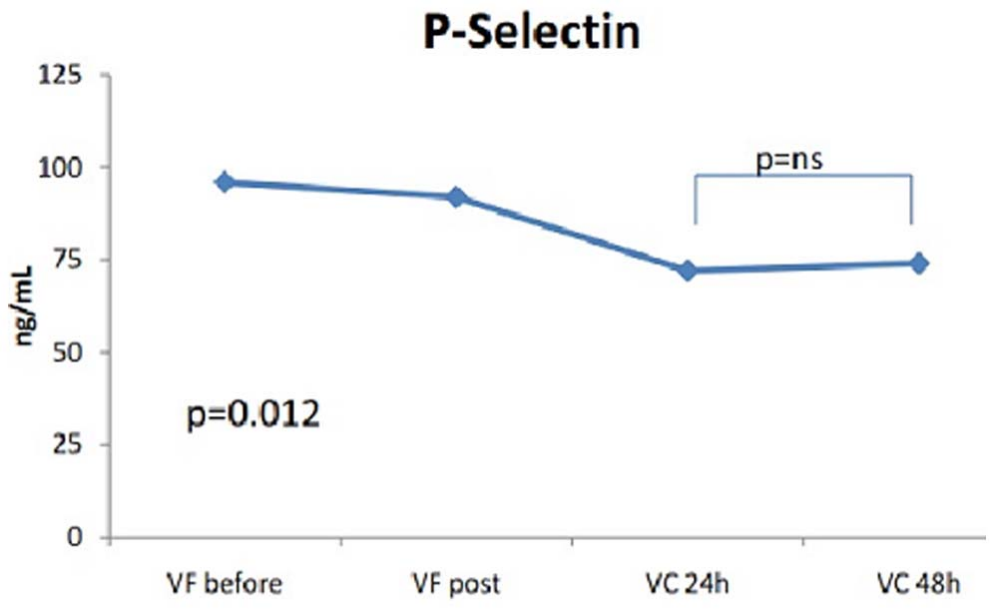
Changes in soluble P-selectin levels after catheter ablation.

The levels of vWF and IL6 – but not sP-sel – increased significantly 24 h after procedure (vWF_pre_ 32 [20–61] vs vWF_post_ 36 [19–55] vs vWF_post_24h_ 44 [29–87], p<0.001, and IL6_pre_ 1.6 [0.9–2.8] vs IL6_post_ 4.3 [1.9–5.9] vs IL6_post_24 h_ 11.3 [6.0–16.5], p<0.001, Figure 1, Figure 2, Figure 3). Persistent AF (Beta = .250, p = 0.031) and LAA flow velocity (Beta = .386, p<0.001) were associated with vWF in cardiac blood after ablation (**Table 5**). Similar as with baseline IL6 levels, advanced age was significantly associated with IL6 levels after ablation in both peripheral and cardiac blood (**Table 6**). In contrast to baseline plasma marker levels, there was significant correlation between vWF and IL6 levels in cardiac blood (r^2^ = .341, p = 0.002) directly after AF ablation while the correlation in peripheral blood did not reach significance (r^2^ = .199, p = 0.079).

**Table 5:** Association between clinical and procedural characteristics with von Willebrandt factor levels after ablation.

Variables	vWF _FV_post_				vWF _LA_post_			
	UV		MV		UV		MV	
	Beta	*P*-value	Beta	*P*-value	Beta	*P*-value	Beta	*P*-value
Age	−.057	0.620			.116	0.307		
Females	−.198	0.081	−.170	0.128	−.028	0.805		
Persistent AF	.274	0.015	.228	0.056	.343	0.002	.250	0.031
LA diameter	.177	0.119	.090	0.463	.192	0.089	.173	0.134
Spontaneous contrast	.151	0.184	.142	0.210	.371	0.001	.386	<0.001
Heparin*	−.060	0.599			−.079	0.492		
Low voltage areas	.013	0.911			.090	0.431		
Cardioversion	.102	0.372			.150	0.188	.006	0.957
Ablation time	.084	0.471			.047	0.690		
Ablation power	.119	0.304			.050	0.670		

**Abbreviations**: UV – univariable analysis, MV – multivariable analysis, CAD – coronary artery disease, PAD – peripheral artery disease, LA – left atrial, EF – ejection fraction, LAA – left atrial appendage, SC – spontaneous contrast, Heparin* - peri-procedural anticoagulation.

**Table 6:** Association between clinical and procedural characteristics with interleukin-6 levels after ablation.

Variables	hsIL6 _FV_post_				hsIL6 _LA_post_			
	UV		MV		UV		MV	
	Beta	*P*-value	Beta	*P*-value	Beta	*P*-value	Beta	*P*-value
Age	.240	0.033	.282	0.021	.241	0.032	.249	0.046
Females	−.013	0.910			.122	0.285		
Persistent AF	.207	0.067	.021	0.867	.229	0.042	.052	0.690
BMI	.157	0.167	.122	0.286	.208	0.066	.182	0.125
LA diameter	.315	0.005	.260	0.069	.214	0.059	.149	0.308
Spontaneous contrast	−.128	0.261			−.110	0.334		
Low voltage areas	.194	0.086	.052	0.686	.152	0.181	.063	0.633
Cardioversion	.202	0.074	−.039	0.757	.226	0.046	.061	0.643
Ablation time	.265	0.021	.266	0.530	.166	0.152	.199	0.647
Ablation power	.254	0.027	−.101	0.804	.154	0.184	−.150	0.720

There were no clinical, procedural or anti-coagulation characteristics associated with post-procedural sP-sel levels in cardiac blood, but only persistent AF remained significantly associated with sP-sel levels in peripheral blood (Beta = −.262, p = 0.020; **Table 7**).

**Table 7:** Association between clinical and procedural characteristics with soluble p-Selectin levels after ablation.

Variables	sP-sel _FV_post_				sP-sel _LA_post_			
	UV		MV		UV		MV	
	Beta	*P*-value	Beta	*P*-value	Beta	*P*-value	Beta	*P*-value
Age	−.117	0.306			−.131	0.251		
Females	−.043	0.705			−.060	0.600		
Persistent AF	−.295	0.008	−.262	0.020	−.152	0.182	−.141	0.264
LA diameter	−.116	0.310			−.193	0.089	−.238	0.068
Spontaneous contrast	−.192	0.090	.175	0.180	−.018	0.872		
Heparin*	−.149	0.189	−.102	0.359	−.072	0.527		
Low voltage areas	.029	0.801			.161	0.156		
Cardioversion	−.084	0.460			−.040	0.728		
Ablation time	−.073	0.533			.154	0.185	.275	0.510
Ablation power	−.070	0.547			.154	0.184	.009	0.983

**Abbreviations**: as in Tables 5–6.

Ablation effects on trans-pulmonary and intra-cardiac gradients in PAF and PersAF are presented in supplemental files (**Tables S1, Figure S1-S3 in File S1**).

### Sub-acute changes in plasma marker levels

In sub-group of 10 patients (age 57±13 years, 70% females) dynamic changes in plasma marker levels were measured in peripheral blood within 2 days after procedure (FV pre-ablation and post-ablation, CV 24 h and 48 h after procedure). Compared to sP-sel and vWF levels 24 h after procedure, the biomarker levels taken 48 h after procedure remained unchanged (**Figure 1**). In contrast, IL-6 levels decreased significantly at 48 h post-procedure compared to levels at 24 h and reached the levels seen directly after procedure.

## Discussion

In this study we hypothesized increase of pro- inflammatory, pro-thrombotic and markers of endothelial damage in peripheral *and* cardiac blood after AF ablation and analyzed the association between clinical, echocardiographic and procedural parameters with these markers. We found that the levels of vWF and IL6 correlated and increased significantly immediately after catheter ablation. Second, vWF levels in cardiac blood were associated with persistent AF and rheological LAA pattern after AF ablation. Finally, while sub-acute vWF levels remained constantly increased, IL6 significantly decreased already 2 days after procedure.

### Catheter ablation and inflammation

Several lines of evidence support a strong association between inflammation and the pathogenesis of AF. Furthermore, inflammatory markers have been investigated in different settings, focusing on their role in prediction of AF occurrence after CABG [10], success of cardioversion [11] or catheter ablation [3].

Several studies suggest that the complement system may serve as a link between inflammation and thrombosis and reveal specific interactions between complement proteins and platelets. Pro-inflammatory cytokines, as IL6, promote thrombosis by increasing expression of different pro-thrombotic markers (e.g. tissue factor, factor VIII and vWF) [12]. These have been linked to increased platelet aggregation, endothelial activation and endothelial cell damage. Moreover, increased IL6 levels lower the concentration of the natural inhibitors of hemostasis as antithrombin and protein S [13]. In this study we demonstrate a 2-fold increase of IL6 levels in both cardiac and peripheral blood directly after procedure followed by 10-fold increase of this marker in peripheral circulation 24 h after procedure. Thus, this might contribute to modulation of later outcomes, e.g. occurrence of thromboembolic complications.

Indeed, we found significant correlations between vWF and IL-6 levels in cardiac circulation after ablation. In contrast to previous study [14], where prolonged inflammatory response for several weeks after ablation was described, we found significant IL6 reduction 48 h after procedure (p<0.001), but still reaching the post-ablative IL6 levels. This dynamic change within 2 days after AF ablation might be partly explained by short half-life of IL6 since it is considered as acute phase protein.

### Endothelial damage/dysfunction

Endothelial damage/dysfunction is one of the pro-thrombotic mechanisms of Virchow's triad [15]. Previous studies have reported increased endocardial expression of vWF in association with thrombus formation in the left atrial appendage (LAA) in patients with AF [16]. This supports the importance of endothelial damage/dysfunction as a plausible mechanism of increased thrombus formation. Although we did not find any association between baseline vWF levels and spontaneous contrast, for the first time we demonstrate that in therapeutically anticoagulated patients LAA flow pattern relates to cardiac vWF levels directly after AF ablation. This finding implicates the necessity of careful anticoagulation during procedure and continuous control thereafter in such group of patients.

The impact on endothelial damage caused by AF catheter ablation is well known [2], [4]. Bulava et al [8] showed changes after catheter ablation on pro-thrombotic and endothelial damage markers, and we confirm their finding showing significantly increased vWF directly after procedure. In contrast to previous study, vWF levels in our study reached their maximum after 24 h, and remained unchanged 48 h after procedure suggesting prolonged endothelial dysfunction and increased risk for early thromboembolic events within first 48 h. In a recent study [17] we demonstrated that majority of post-interventional thromboembolic complications occurred within 2 days after catheter ablation. Prolonged increase of vWF levels 48 h after procedure found in our study indicates possible pathophysiologic relationship between endothelial damage with TE occurrence directly after catheter ablation. Of note, there were no peri-procedural TE in current study, however, this might be related to relatively small patient numbers in the current study.

### Platelet activation

AF is associated with pro-thrombotic state as demonstrated by higher levels of different hemostatic indices [18], [19], [20]. Despite known multiple links between thrombogenesis and inflammation [12] there were no significant correlations between sP-sel and other plasma markers as demonstrated by other studies [20]. As already demonstrated in the SPAF III trial, where several atherosclerotic factors, e.g. male gender, hypertension, peripheral artery disease and diabetes were associated with elevated sP-sel levels [16], in our study baseline sP-sel levels in peripheral blood were significantly associated with hypertension. However, we failed to demonstrate an association between LAA flow pattern and sP-sel levels, as has been shown previously [13].

In contrast to other studies [21] the sP-sel levels were very low in our study population. As possible explanation would be continuous oral anticoagulation before and after procedure (each procedure was performed without cessation of vitamin K antagonists or NOACs) as well as peri-procedural ACT-driven anticoagulation with heparin during ablation. Although we did not find any differences between oral anticoagulation regimes (NOACs/VKA before procedure or with peri-procedural heparin) and sP-sel this might be biased by initially low sP-sel levels.

### Clinical implications

In current study we demonstrated significant increase of vWF and IL6 in peripheral blood within 2 days after procedure which might be partly explained by wash-out phenomenon. Interestingly, while IL6 decreased significantly already 48 h after procedure, vWF remained constant at that time. Our findings support the hypothesis that post-procedural inflammatory response might be a driver of pro-thrombotic reactions facilitating the increased occurrence of peri-interventional thromboembolic events [17]. Although continuous monitoring of anticoagulation before, during and directly after procedure is a standard in current AF ablation management, for the first time our study demonstrates that changes in plasma marker profiles support this clinical approach.

### Limitations

Because of continuous anticoagulation of our study population we found very low sP-sel levels and failed to demonstrate significant sP-sel changes with catheter ablation.

The time of dynamic changes related to catheter ablation was limited by 24 h and in a smaller part by 48 hours. Whether the pattern of these changes is different during longer period after ablation should be investigated in further larger studies. All patients in current study underwent *radiofrequency* catheter ablation. Thus, although some studies demonstrated similar levels of plasma marker changes due to cryoablation or duty-cycled bipolar and unipolar radiofrequency pulmonary vein ablation catheter (PVAC), our findings cannot be directly compared with other AF ablation modalities [22], [23].

### Conclusions

Increase of peripheral vWF and IL6 levels after catheter ablation supports the necessity of continuous and careful control of post-procedural anticoagulation to avoid ablation-related thromboembolic complications.

## Supporting Information

File S1 **File includes Methods, Table S1, and Figures S1-S3.** Figure S1: Effects of catheter ablation on sP-sel levels. A: Peripheral circulation. B: Cardiac circulation. Figure S2: Effect of catheter ablation on hsIL-6 levels. A: Peripheral circulation. B: Cardiac circulation. Figure S3: Effect of catheter ablation on vWF levels. A: Peripheral circulation. B: Cardiac circulation. Methods: Radiofrequency catheter ablation. Table S1: Effects of catheter ablation on plasma markers in different AF types. A: sP-selectin. B: Von Willebrand factor. C: IL-6.
(ZIP)
