# Supplementary figures and images for: Effects of Radiofrequency Catheter Ablation of Atrial Fibrillation on Soluble P-Selectin, Von Willebrand Factor and IL-6 in the Peripheral and Cardiac Circulation
Source: PLoS One. 2014 Nov 12;9(11):e111760. doi: 10.1371/journal.pone.0111760 (PMC4229097; doi:10.1371/journal.pone.0111760)

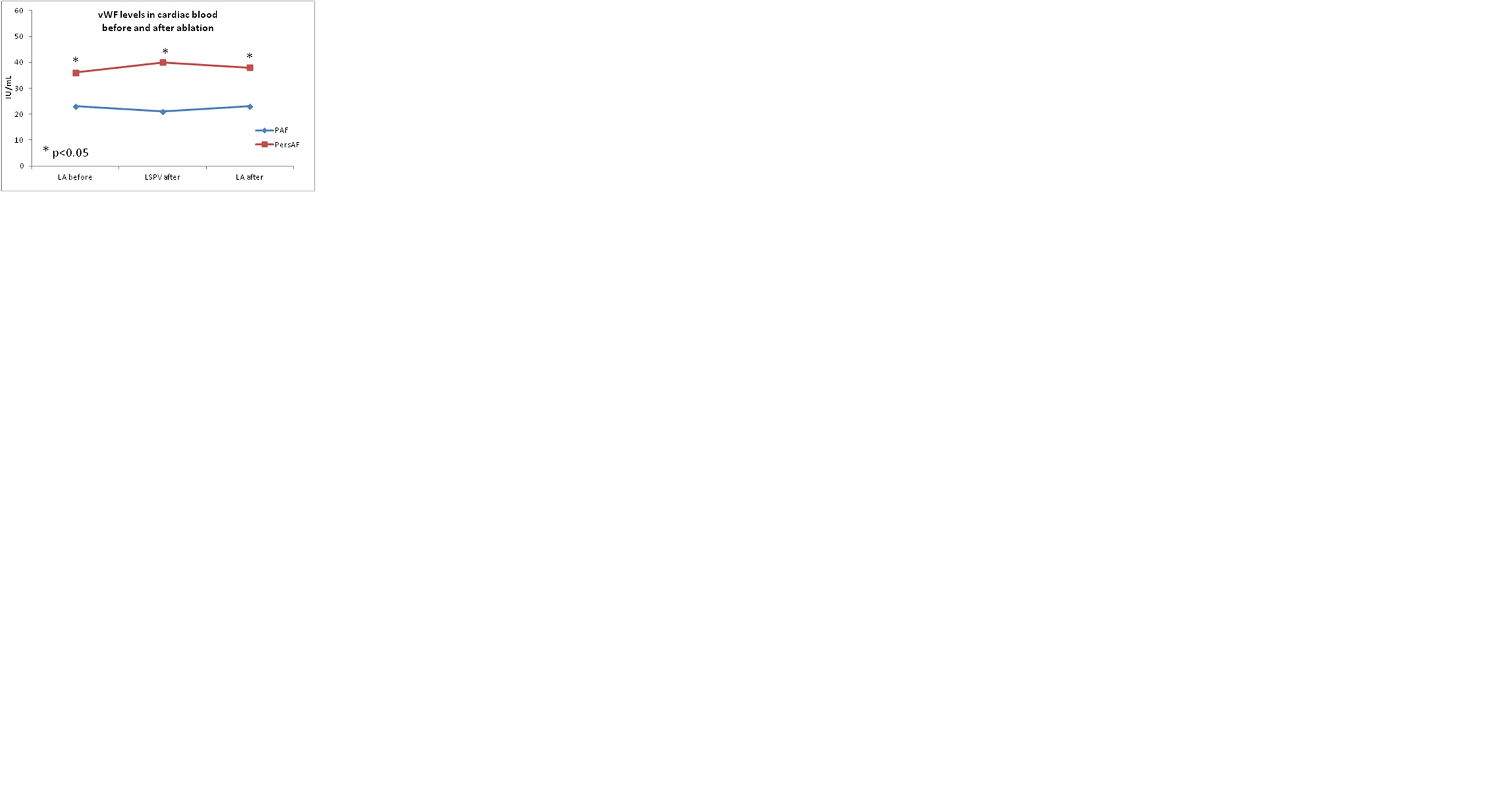

Supplement: File S1 — File includes Methods, Table S1, and Figures S1-S3. Figure S1: Effects of catheter ablation on sP-sel levels. A: Peripheral circulation. B: Cardiac circulation. Figure S2: Effect of catheter ablation on hsIL-6 levels. A: Peripheral circulation. B: Cardiac circulation. Figure S3: Effect of catheter ablation on vWF levels. A: Peripheral circulation. B: Cardiac circulation. Methods: Radiofrequency catheter ablation. Table S1: Effects of catheter ablation on plasma markers in different AF types. A: sP-selectin. B: Von Willebrand factor. C: IL-6. (ZIP) [file pone.0111760.s001.zip › supporting infromation/Figure S3B.tif]

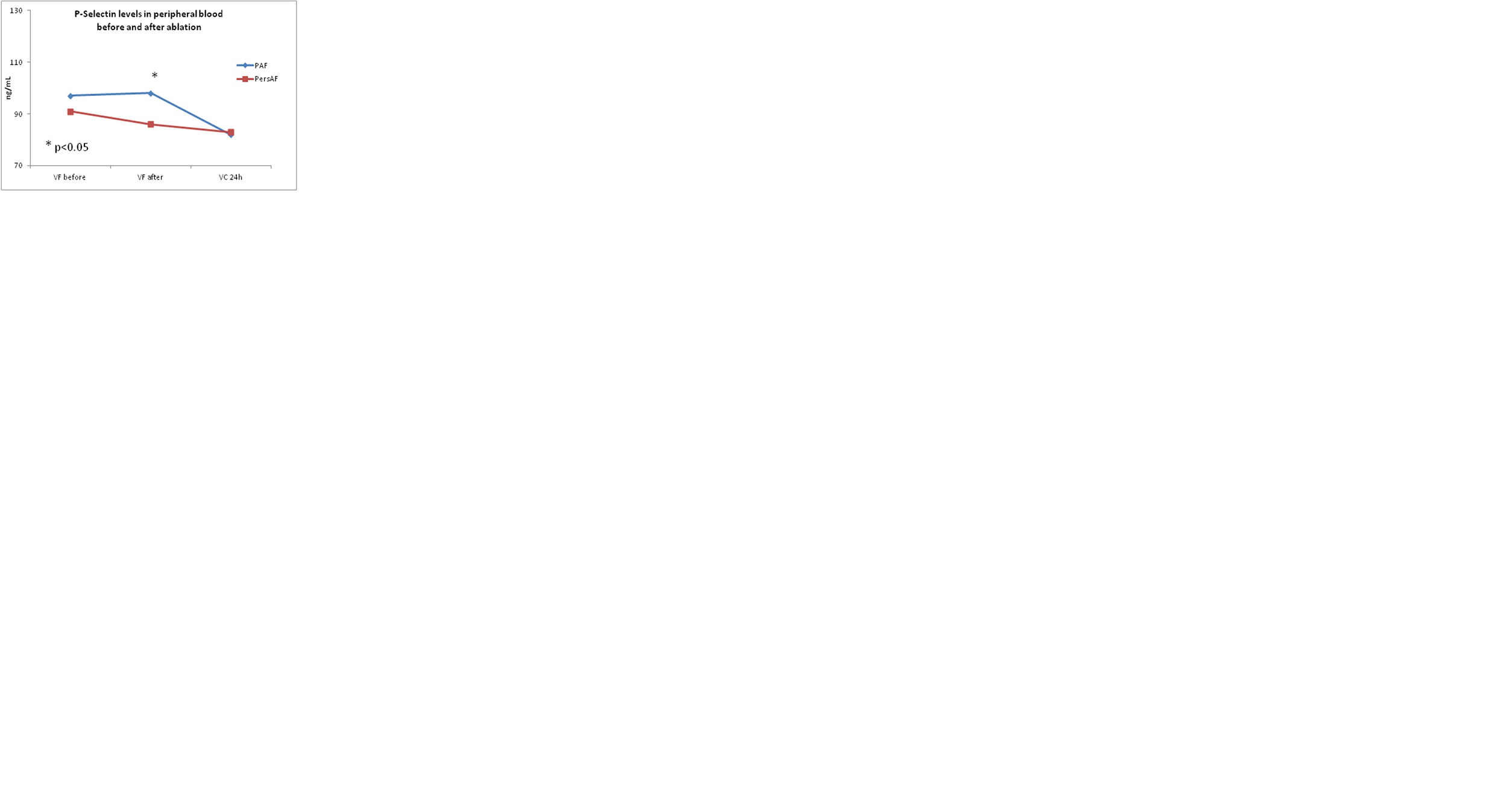

Supplement: File S1 — File includes Methods, Table S1, and Figures S1-S3. Figure S1: Effects of catheter ablation on sP-sel levels. A: Peripheral circulation. B: Cardiac circulation. Figure S2: Effect of catheter ablation on hsIL-6 levels. A: Peripheral circulation. B: Cardiac circulation. Figure S3: Effect of catheter ablation on vWF levels. A: Peripheral circulation. B: Cardiac circulation. Methods: Radiofrequency catheter ablation. Table S1: Effects of catheter ablation on plasma markers in different AF types. A: sP-selectin. B: Von Willebrand factor. C: IL-6. (ZIP) [file pone.0111760.s001.zip › supporting infromation/Figure S1A.tif]

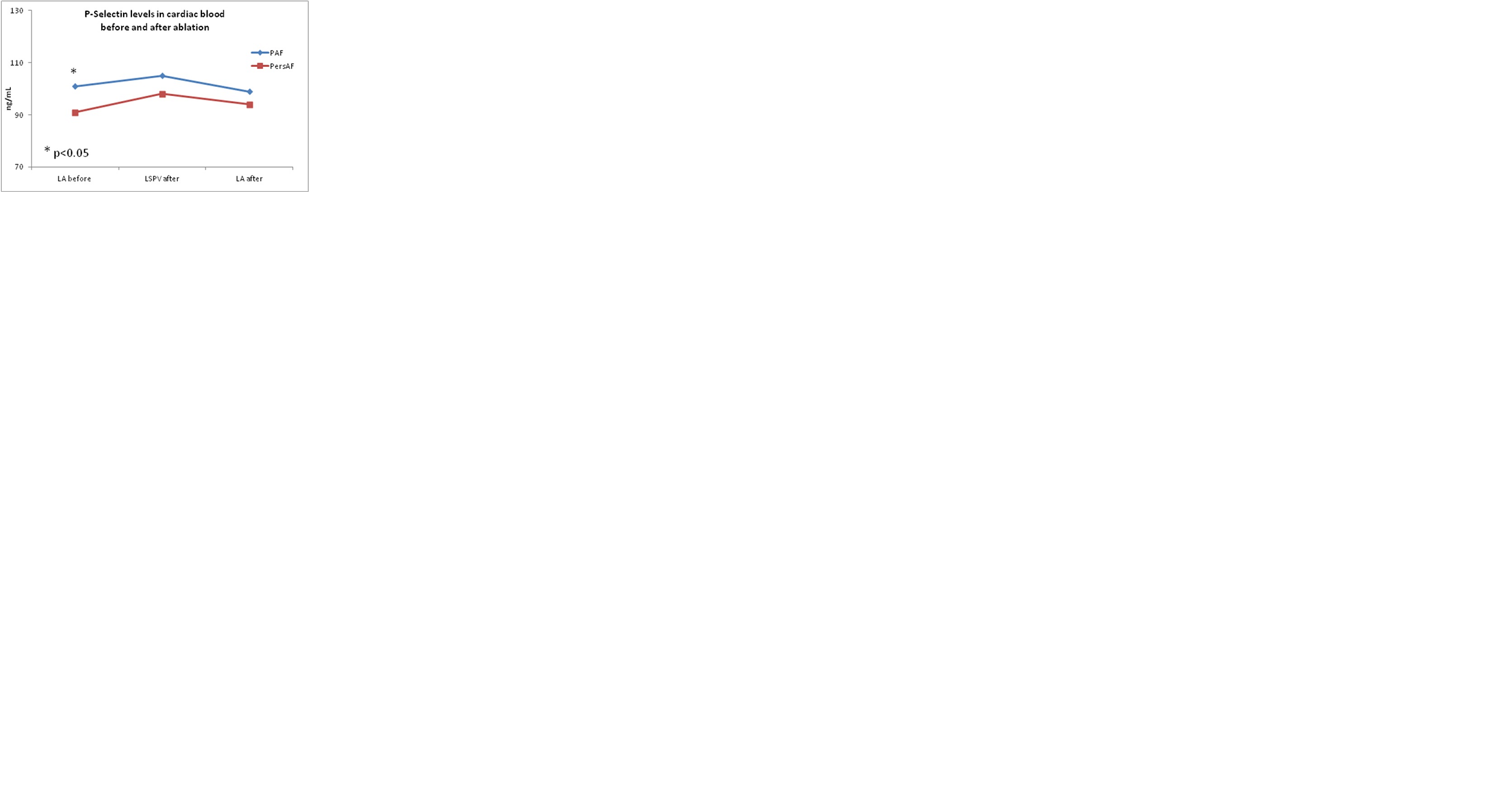

Supplement: File S1 — File includes Methods, Table S1, and Figures S1-S3. Figure S1: Effects of catheter ablation on sP-sel levels. A: Peripheral circulation. B: Cardiac circulation. Figure S2: Effect of catheter ablation on hsIL-6 levels. A: Peripheral circulation. B: Cardiac circulation. Figure S3: Effect of catheter ablation on vWF levels. A: Peripheral circulation. B: Cardiac circulation. Methods: Radiofrequency catheter ablation. Table S1: Effects of catheter ablation on plasma markers in different AF types. A: sP-selectin. B: Von Willebrand factor. C: IL-6. (ZIP) [file pone.0111760.s001.zip › supporting infromation/Figure S1B.tif]

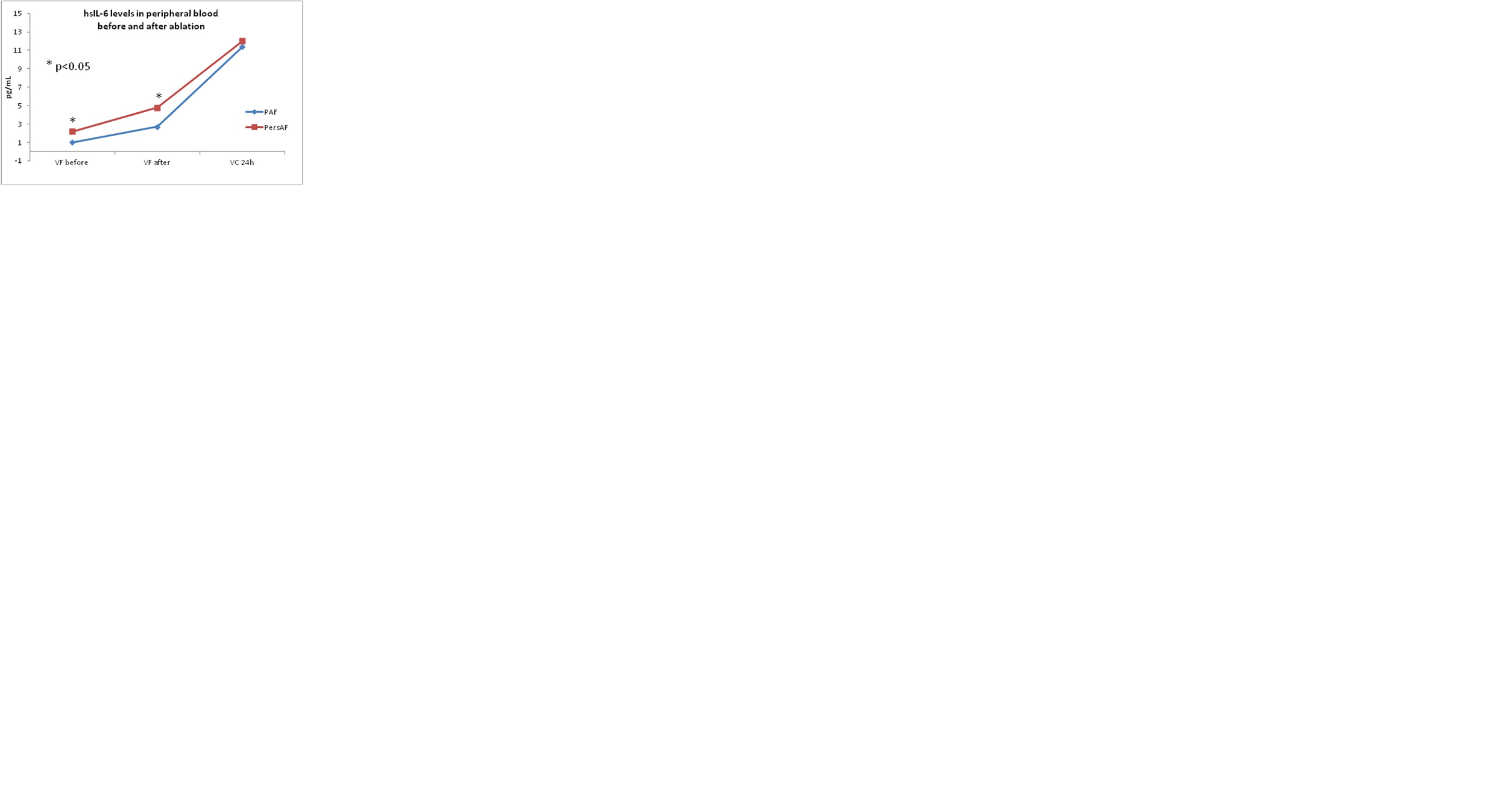

Supplement: File S1 — File includes Methods, Table S1, and Figures S1-S3. Figure S1: Effects of catheter ablation on sP-sel levels. A: Peripheral circulation. B: Cardiac circulation. Figure S2: Effect of catheter ablation on hsIL-6 levels. A: Peripheral circulation. B: Cardiac circulation. Figure S3: Effect of catheter ablation on vWF levels. A: Peripheral circulation. B: Cardiac circulation. Methods: Radiofrequency catheter ablation. Table S1: Effects of catheter ablation on plasma markers in different AF types. A: sP-selectin. B: Von Willebrand factor. C: IL-6. (ZIP) [file pone.0111760.s001.zip › supporting infromation/Figure S2A.tif]

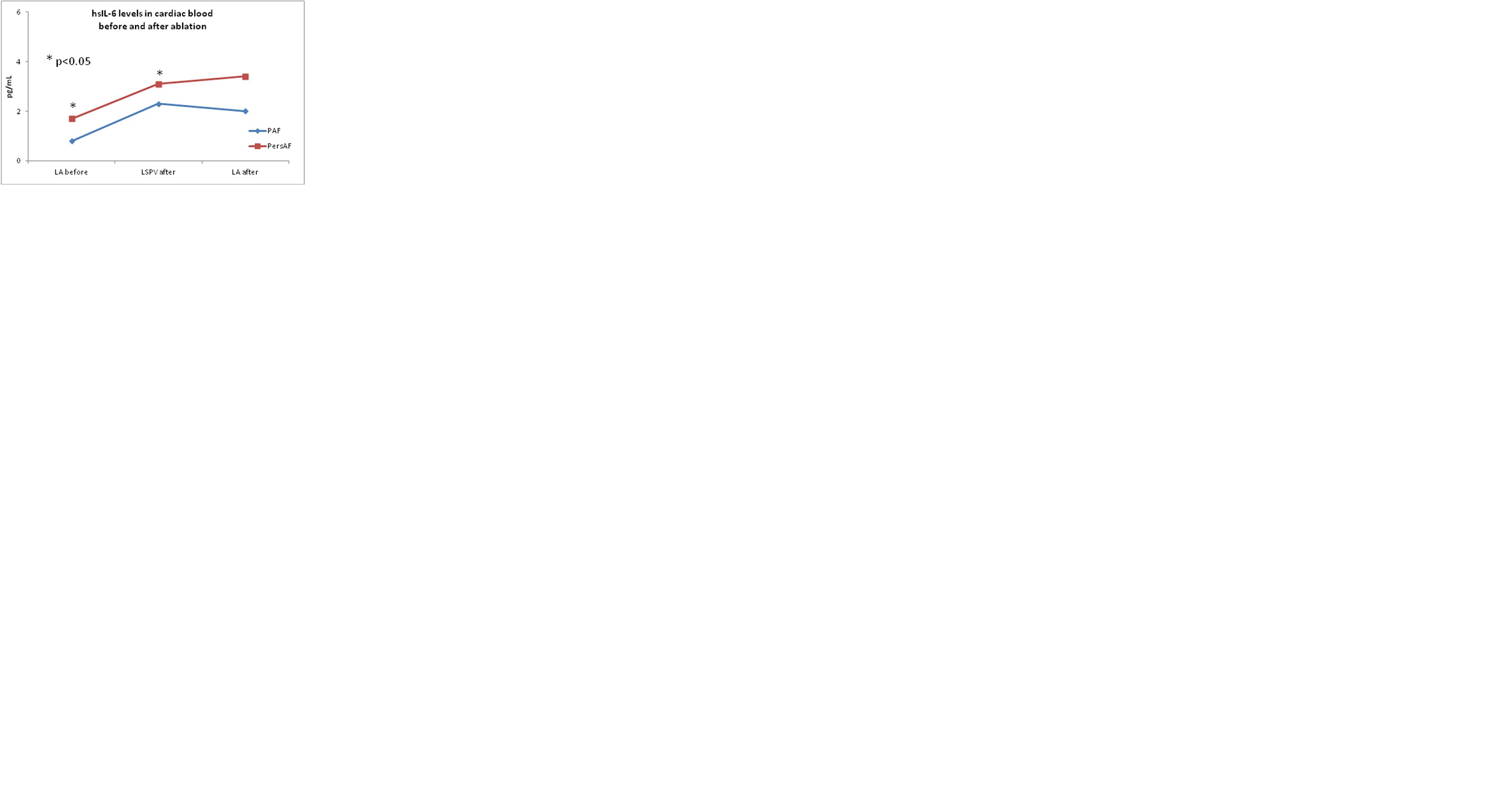

Supplement: File S1 — File includes Methods, Table S1, and Figures S1-S3. Figure S1: Effects of catheter ablation on sP-sel levels. A: Peripheral circulation. B: Cardiac circulation. Figure S2: Effect of catheter ablation on hsIL-6 levels. A: Peripheral circulation. B: Cardiac circulation. Figure S3: Effect of catheter ablation on vWF levels. A: Peripheral circulation. B: Cardiac circulation. Methods: Radiofrequency catheter ablation. Table S1: Effects of catheter ablation on plasma markers in different AF types. A: sP-selectin. B: Von Willebrand factor. C: IL-6. (ZIP) [file pone.0111760.s001.zip › supporting infromation/Figure S2B.tif]

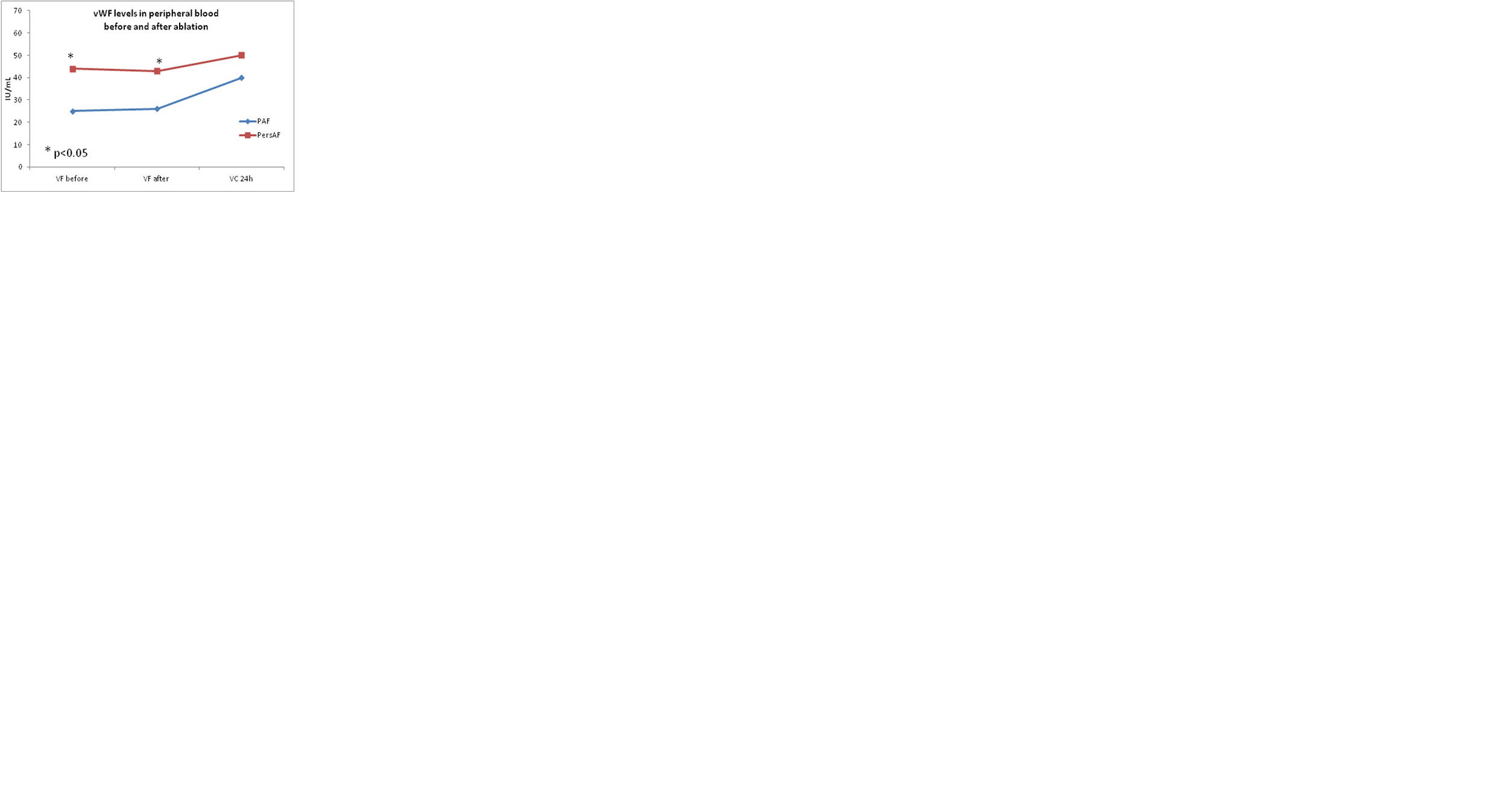

Supplement: File S1 — File includes Methods, Table S1, and Figures S1-S3. Figure S1: Effects of catheter ablation on sP-sel levels. A: Peripheral circulation. B: Cardiac circulation. Figure S2: Effect of catheter ablation on hsIL-6 levels. A: Peripheral circulation. B: Cardiac circulation. Figure S3: Effect of catheter ablation on vWF levels. A: Peripheral circulation. B: Cardiac circulation. Methods: Radiofrequency catheter ablation. Table S1: Effects of catheter ablation on plasma markers in different AF types. A: sP-selectin. B: Von Willebrand factor. C: IL-6. (ZIP) [file pone.0111760.s001.zip › supporting infromation/Figure S3A.tif]
