# Supplementary material for: Effects of Radiofrequency Catheter Ablation of Atrial Fibrillation on Soluble P-Selectin, Von Willebrand Factor and IL-6 in the Peripheral and Cardiac Circulation
Source: PLoS One. 2014 Nov 12;9(11):e111760. doi: 10.1371/journal.pone.0111760 (PMC4229097; doi:10.1371/journal.pone.0111760)
Supplement: File S1 — File includes Methods, Table S1, and Figures S1-S3. Figure S1: Effects of catheter ablation on sP-sel levels. A: Peripheral circulation. B: Cardiac circulation. Figure S2: Effect of catheter ablation on hsIL-6 levels. A: Peripheral circulation. B: Cardiac circulation. Figure S3: Effect of catheter ablation on vWF levels. A: Peripheral circulation. B: Cardiac circulation. Methods: Radiofrequency catheter ablation. Table S1: Effects of catheter ablation on plasma markers in different AF types. A: sP-selectin. B: Von Willebrand factor. C: IL-6. (ZIP) [file pone.0111760.s001.zip › supporting infromation/Table S1C.doc]

**Table S1.** Effects of catheter ablation on plasma markers in different AF types

**C. Interleukin-6**

|  | **PAF** | | **PersAF** | |
| --- | --- | --- | --- | --- |
|  |  | ***P*-value** |  | ***P*-value** |
| **Transpulmonary gradients** |  |  |  |  |
| FV vs LA (before) | 1.0 [0.5-2.0] vs 0.8 [0.5-1.9] | **0.004** | 2.2 [1.1-3.0] vs 1.7 [0.8-2.5] | **<0.001** |
| FV vs LA (after) | 2.7 [1.3-5.7] vs 2.0 [1.0-3.3] | **<0.001** | 4.6 [2.9-5.9] vs 3.3 [2.2-4.3] | **<0.001** |
| **Intracardiac gradient** |  |  |  |  |
| LSPV vs LA (after)* | 2.3 [0.9-5.1] vs 2.0 [1.0-3.3] | 0.056 | 3.0 [1.9-4.5] vs 3.3 [2.2-4.3] | 0.467 |
| **Ablation effect** |  |  |  |  |
| LA (before) vs LA (after) | 0.8 [0.5-1.9] vs 2.0 [1.0-3.3] | **0.001** | 1.7 [0.8-2.5] vs 3.3 [2.2-4.3] | **<0.001** |
| FV (before) vs FV (after) vs CV 24h | 1.0 [0.5-2.0] vs 2.7 [1.3-5.7] vs 11.4 [5.2-16.0] | **<0.001** | 2.2 [1.1-3.0] vs4.6 [2.9-5.9] vs 11.3 [6.0-16.7] | **<0.001** |
