# Supplementary material for: Effects of Radiofrequency Catheter Ablation of Atrial Fibrillation on Soluble P-Selectin, Von Willebrand Factor and IL-6 in the Peripheral and Cardiac Circulation
Source: PLoS One. 2014 Nov 12;9(11):e111760. doi: 10.1371/journal.pone.0111760 (PMC4229097; doi:10.1371/journal.pone.0111760)
Supplement: File S1 — File includes Methods, Table S1, and Figures S1-S3. Figure S1: Effects of catheter ablation on sP-sel levels. A: Peripheral circulation. B: Cardiac circulation. Figure S2: Effect of catheter ablation on hsIL-6 levels. A: Peripheral circulation. B: Cardiac circulation. Figure S3: Effect of catheter ablation on vWF levels. A: Peripheral circulation. B: Cardiac circulation. Methods: Radiofrequency catheter ablation. Table S1: Effects of catheter ablation on plasma markers in different AF types. A: sP-selectin. B: Von Willebrand factor. C: IL-6. (ZIP) [file pone.0111760.s001.zip › supporting infromation/Supplemental material.doc]

**Methods**

**Radiofrequency catheter ablation**

Non-fluoroscopic 3D catheter orientation, CT image integration, and tagging of the ablation sites were performed using Ensite NavX, Ensite Velocity (St. Jude Medical, St. Paul, MN, USA) or CARTO 3 (Biosense Webster, Diamond Bar, CA, USA). Trans-septal access and catheter navigation were performed with a steerable sheath (Agilis, St. Jude Medical., St. Paul, MN, USA).

Access to the LA was obtained through a single or double transseptal puncture (TSP) using the Brockenbrough needle. The decision for single or double TSP was performed upon operator's discretion. A steerable introducer (Agilis NxT, St Jude Medical, St. Paul, MN, USA) and in cases of double TSP a second non-steerable sheath (Swartz, SL0, St Jude Medical, St. Paul, MN, USA) were introduced in to the LA cavity. Patients without therapeutic anticoagulation at the time of ablation received 5000 UI heparin intravenously before TSP followed by additional heparin boluses during the procedure to maintain an ACT 300-400 sec throughout the ablation. In patients on oral anticoagulation therapy with INR 2 – 3, heparin was given first after the TSP.

We used 4-mm irrigated tip ablation catheters (Thermocool, Biosense Webster; Diamond Bar, CA, USA or Cool Flex, St. Jude Medical, St. Paul, MN, USA) with power settings up to 30 W at the posterior wall and up to 45 W elsewhere to perform the pulmonary vein isolation (PVI). 3D mapping navigation systems (Carto 3; Biosense Webster, Diamond bar, CA, USA or EnSite Velocity; Endocardial Solutions, St. Paul, MN, USA) were used to facilitate the ablation. A 3D geometry of the LA and the pulmonary veins was obtained and subsequently superimposed on a subtracted 3D-CT image of the LA. Pulmonary vein (PV) isolation was performed by sequential application of radiofrequency energy at the antrum of the pulmonary veins. End-point was isolation of the PV with proof of both exit and entrance block. No additional lesions outside the PV areas were initially performed to avoid inadvertent LA scaring as a consequence of RF energy application. In patients with previous ablation completeness of circular lesions was proved using Lasso catheter inserted in each PV. In case of re-conduction, the first step was PV re-isolation. If AF did not terminate during ablation, electrical cardioversion was performed.

After the isolation of the pulmonary veins, electoanatomical voltage maps of the LA body in sinus rhythm were created in each patient using ablation catheter as a roving catheter. Potentials with amplitudes over 0.5 mV were defined as normal, and potentials under 0.2 mV as low-voltage. According to the underlying substrate and induced left atrial macro-reentry tachycardias (LAMRT) additional lines transecting the scar areas to connect with healthy tissue or anatomical obstacles were ablated. The completeness of the lines was proved using differential pacing to demonstrate the existence of bidirectional block.
