# Supplementary material for: Effects of Radiofrequency Catheter Ablation of Atrial Fibrillation on Soluble P-Selectin, Von Willebrand Factor and IL-6 in the Peripheral and Cardiac Circulation
Source: PLoS One. 2014 Nov 12;9(11):e111760. doi: 10.1371/journal.pone.0111760 (PMC4229097; doi:10.1371/journal.pone.0111760)
Supplement: File S1 — File includes Methods, Table S1, and Figures S1-S3. Figure S1: Effects of catheter ablation on sP-sel levels. A: Peripheral circulation. B: Cardiac circulation. Figure S2: Effect of catheter ablation on hsIL-6 levels. A: Peripheral circulation. B: Cardiac circulation. Figure S3: Effect of catheter ablation on vWF levels. A: Peripheral circulation. B: Cardiac circulation. Methods: Radiofrequency catheter ablation. Table S1: Effects of catheter ablation on plasma markers in different AF types. A: sP-selectin. B: Von Willebrand factor. C: IL-6. (ZIP) [file pone.0111760.s001.zip › supporting infromation/Table S1A.doc]

**Table S1.** Effects of catheter ablation on plasma markers in different AF types

A. **P-selectin**

| **Soluble P-selectin** | **PAF** | | **PersAF** | |
| --- | --- | --- | --- | --- |
|  |  | ***P*-value** |  | ***P*-value** |
| **Transpulmonary gradients** |  |  |  |  |
| FV vs LA (before) | 96±20 vs 100±21 | 0.081 | 90±22 vs 91±18 | 0.989 |
| FV vs LA (after) | 98±18 vs 99±15 | 0.486 | 86±20 vs 94±21 | 0.005 |
| **Intracardiac gradient** |  |  |  |  |
| LSPV vs LA (after)* | 105±21 vs 99±15 | 0.941 | 98±22 vs vs 94±21 | 0.968 |
| **Ablation effect** |  |  |  |  |
| LA (before) vs LA (after) | 100±21 vs 99±15 | 0.775 | 91±18 vs vs 94±21 | 0.210 |
| FV (before) vs FV (after) vs CV 24h | 96±20 vs 98±18 vs 81±16 | **<0.001** | 90±22 vs 86±20 vs 84±17 | 0.086 |

**Abbreviations**: FV – femoral vein, LA – left atrium, LSPV – left superior pulmonary vein, CV – cubital vein, before – before first radiofrequency impulse, after – at the end of ablation

*Blood from LSPV was available in 44 patients
