# Supplementary material for: Effects of Radiofrequency Catheter Ablation of Atrial Fibrillation on Soluble P-Selectin, Von Willebrand Factor and IL-6 in the Peripheral and Cardiac Circulation
Source: PLoS One. 2014 Nov 12;9(11):e111760. doi: 10.1371/journal.pone.0111760 (PMC4229097; doi:10.1371/journal.pone.0111760)
Supplement: File S1 — File includes Methods, Table S1, and Figures S1-S3. Figure S1: Effects of catheter ablation on sP-sel levels. A: Peripheral circulation. B: Cardiac circulation. Figure S2: Effect of catheter ablation on hsIL-6 levels. A: Peripheral circulation. B: Cardiac circulation. Figure S3: Effect of catheter ablation on vWF levels. A: Peripheral circulation. B: Cardiac circulation. Methods: Radiofrequency catheter ablation. Table S1: Effects of catheter ablation on plasma markers in different AF types. A: sP-selectin. B: Von Willebrand factor. C: IL-6. (ZIP) [file pone.0111760.s001.zip › supporting infromation/Table S1B.doc]

**Table S1.** Effects of catheter ablation on plasma markers in different AF types

B. Von Willebrand Factor

|  | **PAF** | | **PersAF** | |
| --- | --- | --- | --- | --- |
|  |  | ***P*-value** |  | ***P*-value** |
| **Transpulmonary gradients** |  |  |  |  |
| FV vs LA (before) | 25 [18-34] vs 23 [17-32] | 0.322 | 44 [21-74] vs 37 [24-64] | 0.269 |
| FV vs LA (after) | 26 [18-49] vs 23 [15-31] | **0.037** | 42 [26-62] vs 39 [25-54] | 0.528 |
| **Intracardiac gradient** |  |  |  |  |
| LSPV vs LA (after)* | 21 [18-29] vs 23 [15-31] | 0.906 | 40 [21-64] vs 39 [25-54] | 0.060 |
| **Ablation effect** |  |  |  |  |
| LA (before) vs LA (after) | 23 [17-32] vs 23 [15-31] | 0.442 | 37 [24-64] vs 39 [25-54] | 0.912 |
| FV (before) vs FV (after) vs CV 24h | 25 [18-34] vs 26 [18-49] vs 40 [26-54] | **<0.001** | 44 [21-74] vs 42 [26-62] vs 51 [29-99] | **<0.001** |
